# Effect of Fusion Boundary Microstructure on Flow-Accelerated Corrosion Cracking

**DOI:** 10.3390/ma17092026

**Published:** 2024-04-26

**Authors:** Yajing Wang, Zhe Lyu, Zhisheng Wu, Leijun Li

**Affiliations:** 1 Department of Chemical & Materials Engineering, University of Alberta, Edmonton, AB T6G 1H9, Canada; yajing3587@gmail.com (Y.W.); zlyu1@ualberta.ca (Z.L.); 2 School of Materials Science and Engineering, Taiyuan University of Science and Technology, Taiyuan 030024, China; wuzs1963@hotmail.com

**Keywords:** flow-accelerated corrosion, steam pipe, heat-affected zone, fusion boundary, microstructure

## Abstract

Flow-accelerated corrosion (FAC) preferentially attacks the downstream heat-affected zone of the root-pass weld in steam pipe systems. A detailed characterization identifies the fusion boundary as the initiation location for the attack. Alloying elements are found depleted along the weld fusion boundary, and multiple welding thermal cycles and repetitive austenite-to-ferrite phase transformations result in an increased proportion of grains with Goss {110}<001> texture along the fusion boundary. The synergistic effects of chemical segregation and the Schmid factor may contribute to the preferential initiation of FAC cracks along the root weld fusion boundary, making it the weakest link for FAC attack in steam pipe girth welds.

## 1. Introduction

For steam piping systems constructed from low-alloy high-strength steels, flow-accelerated corrosion (FAC) is a prevalent mode of failure, characterized by the dissolution of the protective oxide layer in the wet steam [1]. The consequential severe deterioration of the metal due to FAC poses the risk of catastrophic outcomes [2,3,4]. A comprehensive understanding of the degradation mechanism is paramount to deploying effective mitigation strategies essential for ensuring the safety of these critical components.

The current understanding of the rate of metal wall thickness loss attributed to FAC entails complex interactions among various parameters, encompassing fluid dynamics, steam chemistry, and metallurgical factors of the piping, such as chemical composition, microstructure, and weld joints. Fluid dynamics parameters, inclusive of flow rate, surface roughness, geometric layout, and steam proportion, influence by modulating the mass transfer rate of corrosion products to the fluid, thereby affecting the FAC rate. Water chemistry serves as a pivotal factor, with low pH, high oxygen content, and the presence of aggressive ions (e.g., chloride and sulfate) hastening the corrosion process. However, a limited number of studies have delved into elucidating the role of metallurgical factors in the FAC mechanism.

At present, three primary methods are utilized for investigating flow-accelerated corrosion: impact jet systems [5,6,7], rotating cylindrical or circular electrode systems [8,9,10,11,12], and loop systems [13,14,15,16,17]. These methodologies are frequently coupled with corrosion test electrodes to assess the overall corrosion rate of specimens. Karlsdottir et al. [18] explored the influence of flow velocity on corrosion rates in superheated geothermal steam. Zhang et al. [13] integrated array electrode technology with computational fluid dynamics (CFD) simulation to establish correlations between corrosion behavior, flow velocity, and shear stress distribution at a 90 deg bend. Li et al. [4] examined the impact of dissolved oxygen and wall shear stress on corrosion inhibitors in pipeline steel using a high-shear turbulent channel flow cell. Utanohara et al. [19] determined the FAC rate downstream from an orifice using a high-temperature water test loop and evaluated the effects of the flow field on FAC through CFD simulations. Ajmal et al. [20] analyzed the FAC rate of intrados and extrados of an API X70 pipe steel elbow in a flow loop system, utilizing CFD to simulate shear stresses and observing an inverse relationship between the corrosion rate and shear stresses. However, challenges emerge in validating results from laboratory-scale simulations due to the limited availability of field data obtained from actual operating conditions. Consequently, the applicability of laboratory findings to industrial pipeline operations remains uncertain.

According to Tomarov et al. [21], FAC damage can be classified into two distinct types: general flow-accelerated corrosion (GFAC) and local flow-accelerated corrosion (LFAC). GFAC, observed in most instances, is characterized by a moderate metal thinning rate that typically does not result in sudden failures or pinholes compromising the circuit’s integrity. In contrast, LFAC entails significant metal thinning in a localized area, potentially leading to the formation of wormholes or sudden pipeline damage. Fundamental disparities exist between GFAC and LFAC regarding influencing factors, consequences, and occurrence locations. Therefore, it is imperative to employ distinct methodologies to investigate the initiation and progression of flow-accelerated corrosion. In the case of LFAC, limited reports exist regarding initial damage, such as pits or cracks. Current research primarily focuses on comprehending the impacts of service environment conditions, flow dynamics, and water chemistry on FAC damage. The influence of metallurgical factors, such as chemical segregation and microstructure, on the onset of local flow-accelerated corrosion has not received adequate attention or comprehensive investigation. LFAC damage is commonly identified in pipe bends (elbows), tube constrictions, and other geometric irregularities such as weld joints, which induce sudden changes in flow direction or velocity [22].

Welding introduces microstructural and compositional heterogeneity across the weld joint. Consequently, weld joints typically exhibit localized effects, necessitating sophisticated experiments to pinpoint specific vulnerable areas. This study explores LFAC cracking initiation in the heat-affected zone of steam pipe girth welds and positively identifies the weld fusion line as a preferred location for flow-accelerated corrosion-induced crack initiation.

## 2. Materials and Methods

In our prior study [23], several weld rings were extracted from different locations in the service-exposed steam pipe. The FAC damage observed in the girth welds exhibited common characteristics, including thinning caused by liquid droplet impingement primarily concentrated on the root weld protruding reinforcement; FAC microcracks originating at the fusion line and propagating in the coarse-grained heat-affected zone of the root weld; and FAC damage concentrated at the downstream root weld toe. This paper focuses on developing a rationale for why the weld fusion line has been the crack initiation point for FAC.

The chemical compositions of the base metal (BM) and root-pass weld metal (WM) are listed in Table 1. The root-pass weld used shielded-metal arc welding, delivering 34 kJ/in heat input with the E6010 electrode. The filling and cap passes were made using the E8010 electrode for flux-cored arc welding, delivering 57 kJ/in heat input. A minimum preheating and interpass temperature of 150 °C was maintained during welding.

Sections of corrosion-attacked pipes were extracted and analyzed in a previous report [23]. For this study, the specimen that showed crack initiation was investigated further. For metallographic observations, standard sample preparation procedures were followed. The samples underwent mounting, grinding, and polishing using the Buehler (Lake Bluff, IL, USA) Ecomet 250/300 grinder–polisher. Grit size #180, #320, #600, #800, and #1200 SiC abrasive papers were successively used for grinding, followed by polishing with 3.0 μm, 1.0 μm, and 0.5 μm diamond suspensions. The sample surface was etched using a 4% nital solution. For electron back-scattered diffraction (EBSD) characterization, the specimen was additionally polished using a 0.05 μm alumina suspension and a 0.02 μm colloidal silica solution.

The Zeiss (Toronto, ON, Canada) Stemi 508 stereo microscope and Olympus (Tokyo, Japan) LEXT OLS3000 confocal laser scanning microscope (CLSM) were used for surface morphology at lower magnifications. Additionally, the Zeiss (Toronto, ON, Canada) Sigma Field Emission Scanning Electron Microscope (FE-SEM) was used for the microstructure. Electron back-scattered diffraction (EBSD) characterizations were performed within the same FE-SEM vacuum chamber, with a scanning step size ranging from 150 to 400 nm. For phase selection and pre-definition ahead of detection, HKL (HKL Research Inc.) and ICSD (Inorganic Crystal Structure Database) were utilized. Post-processing of EBSD data was conducted using Oxford (Ulm, Germany) AZtecCrystal and the open-source MATLAB MTEX toolbox for crystal visualization [24]. Electron probe microanalysis (EPMA) was conducted on a JEOL (Tokyo, Japan) JXA-8900R electron microprobe, equipped with five tunable wavelength-dispersive spectrometers. Operating conditions included a 40-degree tilt angle, a beam energy of 20 keV accelerating voltage, a beam current of 300 nA, and a beam diameter of 0.1 μm. It should be noted that the quantitative results for Nb and Cr were not collected due to their levels being below the EPMA detection limits. Detections were recorded at successive distances of 3 microns across the heat-affected zone and weld metal.

Nano-indentations were conducted using an Anton Paar (Toronto, ON, Canada) Step 300 Surface Testing Platform equipped with a diamond pyramid tip in the linear loading mode with a 100 mN maximum load and a 10 s dwell time. The load–depth curves were analyzed using the Oliver and Pharr method [25,26,27,28,29]. To ensure repeatability, experiments for each region were conducted multiple times to collect the scatter band, with only representative curves shown. Microhardness measurements were conducted using a Tukon (Triadelphia, WV, USA) 2500 Vickers hardness tester, with a 0.05 kgf load and a 10 s dwell time.

To reveal the flow velocity distributions on the inner diameter surface of the weld joint in the pipe, single-phase (water) and dual-phase (steam-water) computational fluid dynamics (CFD) simulations were conducted using COMSOL (Burlington, MA, USA) Multiphysics Version 5.5 and Ansys (Canonsburg, PA, USA) Fluent Version 2020 R2.

## 3. Results

**Figure 1:**
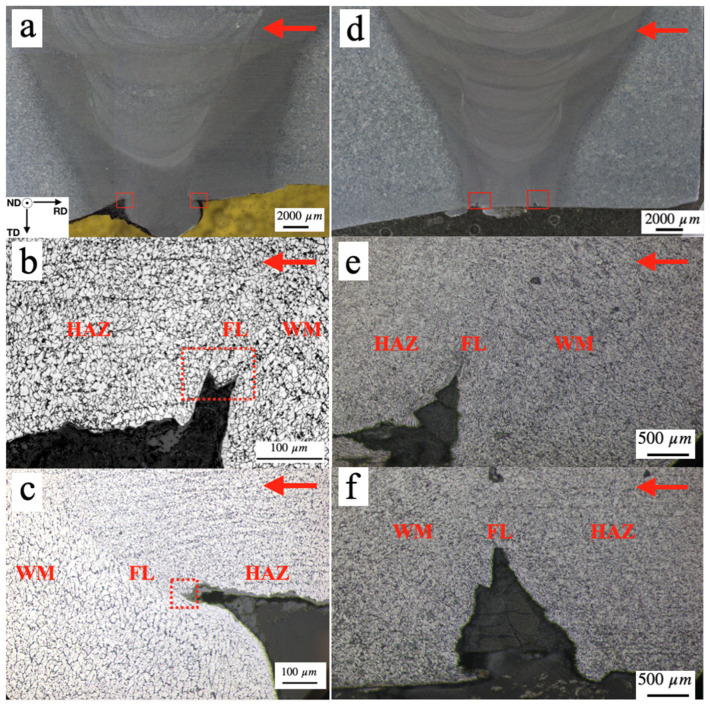
(**a**) Weld joint section from the 12 o’clock location shows crack initiations. The red arrows point to the steam flow direction. (**b**) Crack initiation point at the fusion line near the downstream toe of the root pass, indicated by the red box on the left in (**a**). (**c**) Crack initiation point at the fusion line near the upstream toe of the root-pass weld, indicated by the red box on the right in (**a**). (**d**) Weld joint from the 3 o’clock location. (**e**) Crack initiation point at the fusion line near the downstream toe of the root pass, indicated by the red box on the left in (**d**). (**f**) Crack initiation point at the fusion line near the upstream toe of the root-pass weld, indicated by the red box on the right in (**d**).

Figure 1 shows the micrographs of the girth welds that contained crack initiations for flow-accelerated corrosion attack. The red arrows point to the direction of steam flow. Subsequent characterization used the following frame of reference for the specimen: following the right-hand rule, the X-axis is in the radial direction (RD), the Y-axis in the thickness direction (TD), and the Z-axis in the normal direction (ND). Under this frame of reference, the microcracks were found to have initiated at both upstream and downstream weld toes and propagated along the Y-axis direction, as indicated by the red rectangles in Figure 1a,d. At a higher magnification, the crack initiations were identified at the weld fusion line (FL), for both the downstream (Figure 1b,e) and the upstream (Figure 1c,f) weld toes. No FAC crack initiations were found in the adjacent heat-affected zone (HAZ) or in the root-pass weld filler metal (WM). It is notable that FAC crack initiations were detected at the fusion line of the root pass at various locations of the steam pipe girth weld. The macrograph shown in Figure 1a was captured from the 12 o’clock location of the girth weld, while the macrograph shown in Figure 1d was captured from the 3 o’clock location of the girth weld, assuming the observer was looking at the flow direction. It is apparent that the protruding weld reinforcement at the 3 o’clock location has been severely eroded by the flow; it also contains two small crack tips, each located at the weld fusion line of the upstream and downstream weld toes. The 12 o’clock location, in contrast, has the root weld reinforcement intact, although the crack initiations at the weld fusion line can be observed.

**Figure 2:**
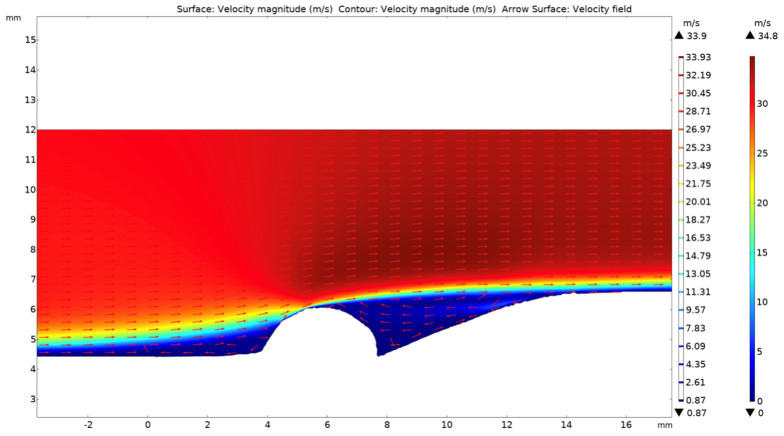
Flow pattern and velocity distribution over the surface of the 12 o’clock weld, computed in Comsol Multiphysics.

Figure 1a was digitized and converted into 2D finite-element meshes for the CFD simulation in COMSOL Multiphysics. A single-phase water film flow with an assumed velocity of 30 m/s is shown in Figure 2. As the flow passes through the girth weld surface, there are disruptions in the flowing water. The relationship between component surface shear stress (τ) and flow velocity (u) can be explained by the following equation [30], $\tau =\mu \frac{\mathrm{du}}{\mathrm{dy}}$, where y is distance, μ is the dynamic viscosity of the water, and du/dy is the slope of the flow velocity distribution curve. Near the protruding root weld reinforcement, a sudden alteration in flow velocity results in an increase in the value of du/dy, leading to higher shear stress and more significant mechanical erosion at the location. Near the fusion line at the weld toe, the water is stagnant, resulting in less fluid erosion.

Additionally, dual-phase CFD simulations were conducted using Ansys Fluent to elucidate the factors contributing to the varying FAC damage observed in different locations along the girth weld. The Eulerian multiphase method was employed to model the vapor/water dual-phase flow, with phase change considered. The geometry was constructed from the measured dimensions of the pipe. At the inlet of the pipe, it was assumed that only the vapor phase was present; the temperature and pressure of the vapor used for simulation were selected as 350 °C and 14,700 kPag. LEE’s phase change model [31] was utilized to simulate the condensation of vapor.

**Figure 3:**
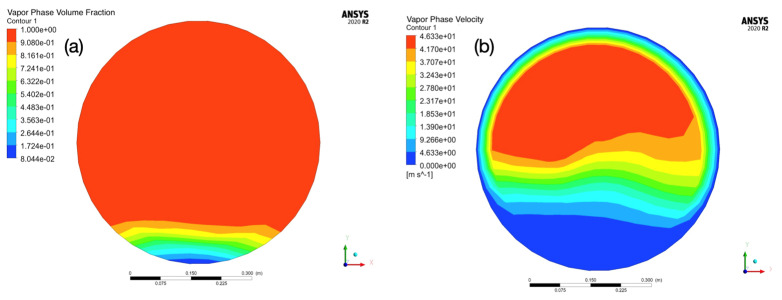
CFD results simulated using Ansys Fluent software. (**a**) depicts the cross-sectional phase distribution diagram of the flow, and (**b**) depicts the cross-sectional flow velocity distribution.

Figure 3 shows the phase and velocity distribution maps of the flowing steam–water mixture. It can be observed that due to heat and pressure loss along the transmission direction, the circulating steam has partly condensed to water. Additionally, influenced by gravity, the condensed water accumulates at the bottom of the pipe, as shown in Figure 3a. Seen in Figure 3b, due to collection of the condensed water, the area of the vapor phase is reduced. Compared to the initial steam velocity at the inlet (30 m/s), the velocity of the vapor phase increases to 46.3 m/s, while the condensed water at the bottom of the pipe is more stagnant, with a velocity of 4.6 m/s. A large velocity difference can generate a shear force at the vapor–liquid interface. If this shear force exceeds the strength of the liquid at the interface, some liquid will be sheared off the liquid layer and be carried along with the rapidly flowing vapor. Some of these sheared droplets may collide with the protruding weld reinforcement. This phenomenon, known as liquid droplet impingement (LDI), rapidly removes the oxide layer on the metal surface, thereby promoting flow-accelerated corrosion.

**Figure 4:**
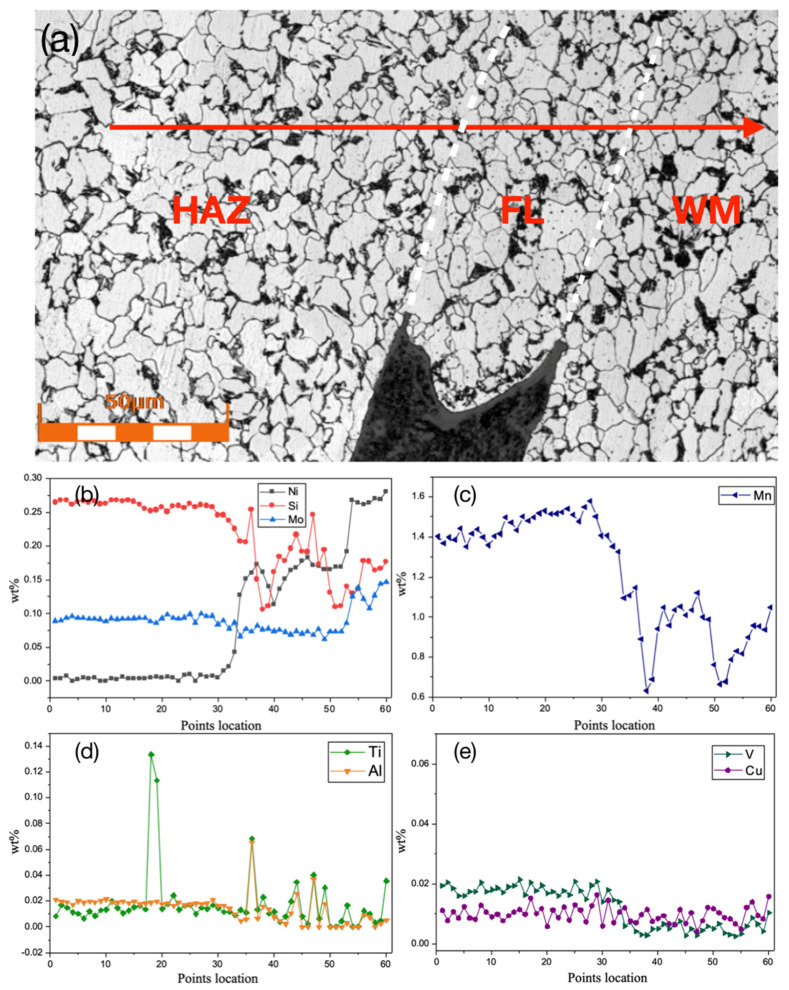
(**a**) The EPMA line scan along the arrow mark across the FL from the HAZ to the WM. (**b**) Concentrations of Ni, Si, and Mo, and (**c**) concentration of Mn. (**d**) Concentrations of Ti and Al, and (**e**) concentrations of V and Cu.

The microstructure and quantitative compositional analysis by EPMA on the downstream crack-tip region are shown in Figure 4a. The fusion boundary or fusion line (FL) is a region several grain sizes wide, sometimes referred to as the partially melted zone. In this region, the peak temperature falls between the solidus and liquidus of the steel. At this magnification, the ferrite grains in the fusion boundary contain some precipitates, as do the weld metal ferrite grains. On the other hand, the ferrite grains in the heat-affected zone do not contain observable precipitates at all. The red arrow indicates the location of the EPMA line scan. Shown in Figure 4b–e, the fusion line region has the average (of the base metal and weld metal) chemical compositions for Ni and Mn, likely due to the dilution by the base metal. There is a depletion of carbide-forming elements (Mo and V) in the fusion line region, although the de-oxidizing elements Ti and Al show several peaks, indicating possible oxide inclusions.

**Figure 5:**
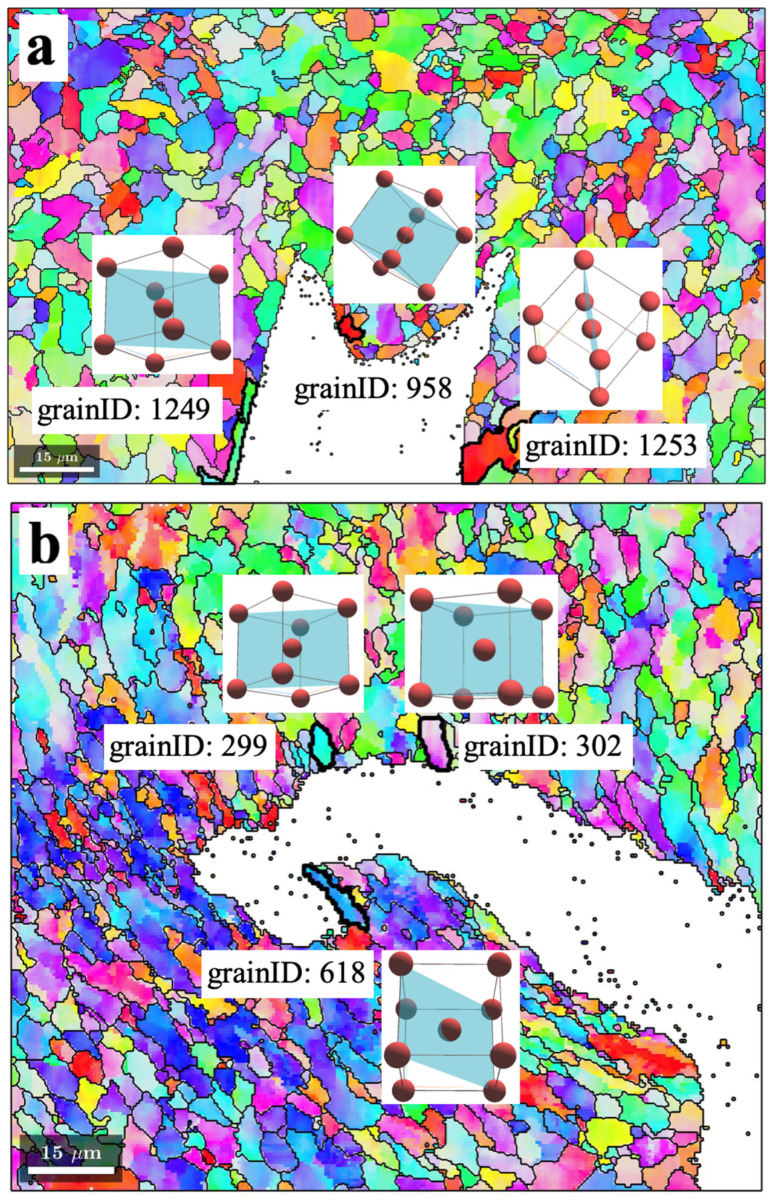
EBSD crystal orientation mapping with respect to the Z-axis of the (**a**) downstream weld toe, and (**b**) the upstream weld toe. Grains outlined by black bold lines were randomly selected for orientation visualization.

Figure 5 shows the EBSD crystal orientation maps with respect to the Z-axis (specimen normal direction) of downstream and upstream crack-tip areas outlined by the rectangles shown in Figure 1b,c. In the downstream fusion boundary (Figure 5a), three grains along the crack path with ID numbers 958, 1249, and 1253 were randomly selected for spatial crystal visualization. The {110} plane family for grains 1249 and 1253 aligned closely with the crack surface. The {110} plane family for grain 958 did not align closely with the crack surface. Similarly, in the upstream fusion line area (Figure 5b), three grains (ID numbers 299, 302, and 618) on the crack path were randomly selected and their corresponding crystal unit cells were visualized. The {110} crystal planes of grains 299 and 302 were found to align with (i.e., be parallel to) the crack surface.

Subsequently, based on the EBSD mapping (Figure 5), the MATLAB MTEX toolbox was employed to calculate and plot the Young’s modulus (in units of GPa) for each analyzed pixel. The modulus is typically insensitive to the material’s microstructure and only depends on the strength of atomic bonding, which is detected by the crystallographic orientation parameters of each pixel. The assessment of Young’s modulus values for anisotropic materials can be achieved by computing the values in specific directions using a given fourth-order stiffness tensor, C_ij_. In this study, for computation in the MTEX program, the fourth-order stiffness matrix of body-centered cubic (ferrite) proposed by Romain [26] was imported:

$$\left[\begin{array}{cccccc} 247 & 150 & 150 & 0 & 0 & 0\\ 150 & 247 & 150 & 0 & 0 & 0\\ 150 & 150 & 247 & 0 & 0 & 0\\ 0 & 0 & 0 & 97 & 0 & 0\\ 0 & 0 & 0 & 0 & 97 & 0\\ 0 & 0 & 0 & 0 & 0 & 97 \end{array}\right]$$

**Figure 6:**
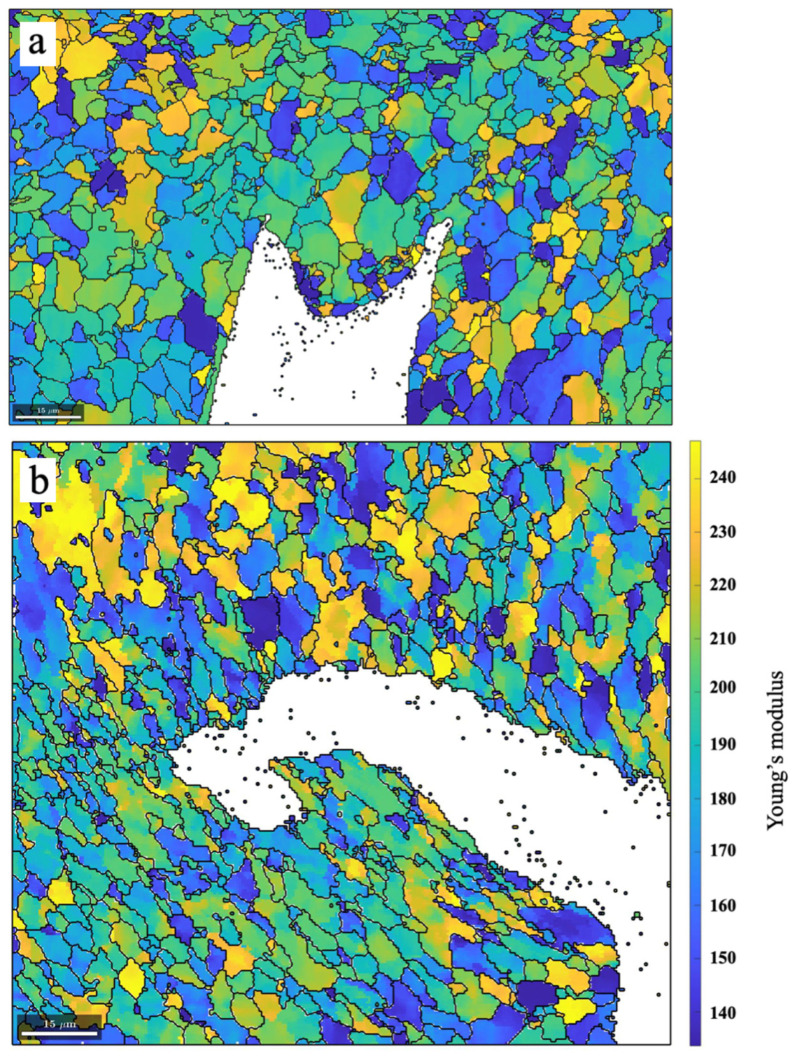
The distribution of Young’s modulus values in the (**a**) downstream and (**b**) upstream crack-tip areas, obtained by specifying the direction of external force aligned with the Y-axis in MTEX software.

By specifying the direction of external force aligned with the steam pressure direction (e.g., the Y-axis) of the girth weld in MTEX (London, UK) Version 5.11 software, the distribution of Young’s modulus values in the upstream and downstream crack-tip areas was obtained (Figure 6). It is evident that the fusion line is weaker than the adjacent base metal and weld metal. The FAC crack initiations appear to preferentially propagate along the weld fusion line with a lower Young’s modulus.

**Figure 7:**
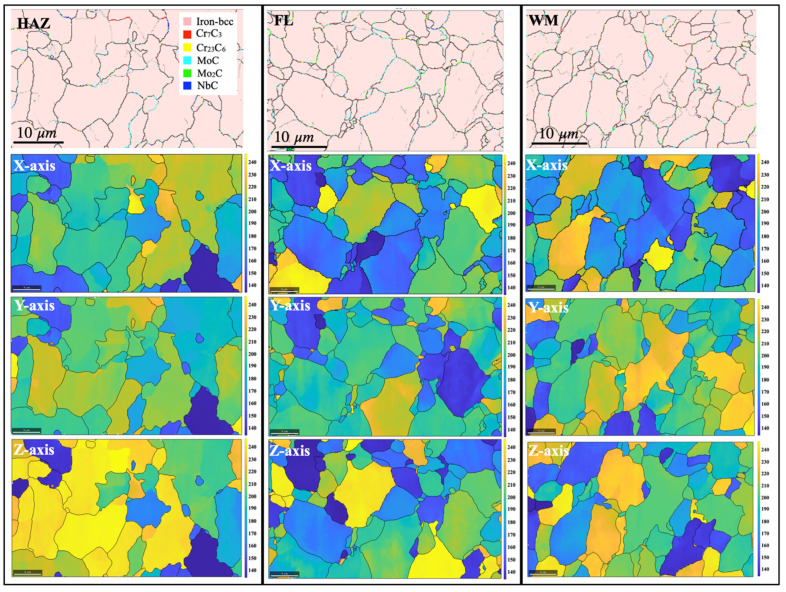
EBSD phase maps for the heat-affected zone (HAZ), fusion line (FL), and weld metal (WM) overlaid with grain boundaries, and the corresponding Young’s modulus maps calculated along the X-, Y-, and Z-axes, respectively. The different colors can be understood by referring to the legends.

EBSD results measured with a finer step size of 0.15 μm from the heat-affected zone, fusion line, and weld metal adjacent to the downstream crack tip are shown in Figure 7. Precipitate quantities, grain sizes, and fractions of high-angle and low-angle grain boundary types are summarized in Table 2. No significant differences in grain sizes or grain boundary types were observed among the heat-affected zone, fusion line, and weld fusion zone. The volume fractions of precipitates were almost identical, except that the fusion line contained more Mo–carbide.

**Figure 8:**
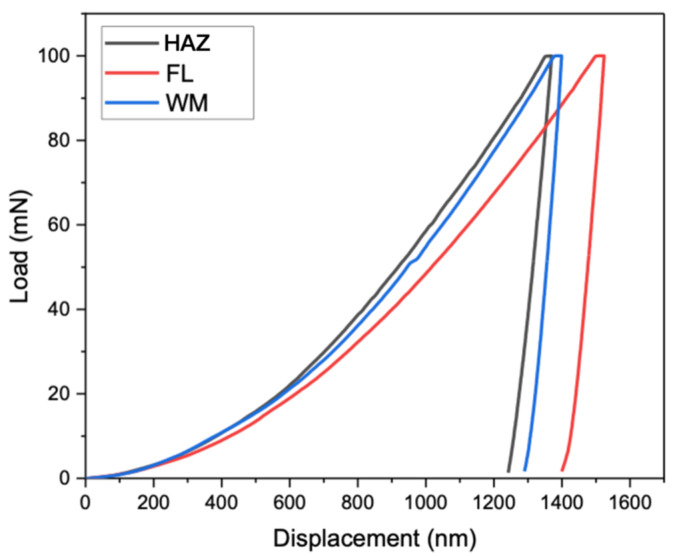
The load–displacement curves for the HAZ, FL, and WM, respectively.

Table 3 lists the Vickers hardness results, Young’s modulus measured from instrumented indentation tests, and Young’s modulus calculated from EBSD mapping for the weld metal, fusion line, and heat-affected zone. The hardness values for the fusion line were the lowest among the three regions. The Young’s modulus values from both the indentation tests and EBSD tests were also the lowest for the fusion line. The load–displacement curves for the three regions, analyzed using the Oliver and Pharr method, are shown in Figure 8. The loading and unloading curve for the fusion line area showed a maximum penetration depth, indicating the lowest deformation resistance along the fusion line, whereas the heat-affected zone and weld metal showed lower displacements, confirming the higher Vicker’s hardness.

## 4. Discussion

The CFD simulation indicates that water is sparse near the 12 o’clock position of the circumferential weld, while water is more densely distributed close to the bottom (6 o’clock) of the circumferential weld. The steam flow exhibits a cross-sectionally non-uniform distribution within the pipe, contributing to the varying FAC damage morphology observed in different locations along the girth weld. Additionally, the flow over the fusion line (or weld toe) areas is more stagnant, indicating that these regions are less affected by erosion. However, the microstructure of the material, such as the distribution of alloying elements and crystallographic orientations, determines the local mechanical properties that respond to the development of FAC cracks at the weld fusion line.

**Figure 9:**
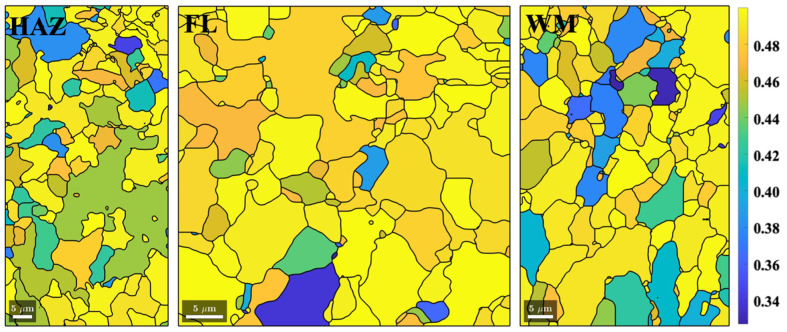
Schmid factor computed for the weld fusion line loading direction. For the heat-affected zone (HAZ), the average Schmid factor is 0.467. For the fusion line region (FL), the average Schmid factor is 0.476. For the weld metal (WM), the average Schmid factor is 0.466.

The crack initiation for flow-accelerated corrosion seems to happen preferentially along the {110} slip plane for the ferritic (BCC) microstructure. The reason for the fusion line being the weakest link for the crack initiation is because the fusion line contains the highest fraction of grains with the {110} planes aligned with it. This argument is supported by evidence shown in Figure 9, which shows the Schmid factor calculated for loading along the weld fusion line direction (or the crack propagation direction). It is evident that the grains in the fusion line region have the highest average Schmid factor (0.476), indicating more favorable slip planes are aligned with the fusion boundary. The larger the Schmid factor (i.e., closer to 0.5), the easier it is for the slip systems to be activated. In Figure 9, the heat-affected zone and weld metal contain a smaller number of grains with the slip systems aligned with the loading direction (i.e., along the fusion line).

The reason why the fusion line region contains the highest fraction of textured grains may be attributed to the characteristics of the fusion line itself. The fusion line region is a partially melted zone where the peak temperature falls between the liquidus and solidus of the molten pool. Upon solidification, the dendritic grains grow epitaxially on top of base metal half-melted grains, with grains whose <100> directions align with the negative of the thermal gradient to grow faster [32]. Since the weld fusion line is perpendicular to the thermal gradient direction, the <100> crystal directions of the cubic system also align with the fusion line (in three dimensions, a plane). As can be shown by a Scheil calculation, the initial dendrites in the partially melted zone contain lower concentrations of alloying elements due to segregation. This explains the reason for the depletion of alloying elements in the weld fusion line region. When structural components are exposed to aggressive environments, any chemical gradient will create favorable conditions for galvanic corrosion. The areas depleted of alloy elements will be preferentially corroded as the anode, while the element-enriched areas will have a slower corrosion rate as the cathode. Local segregation of alloying elements can also affect the cracking of the interfaces. Lee et al. [33] found the crack propagation in high-strength low-alloy steel to be accelerated by the uneven distribution of alloying elements (Mn, Ni, Cr).

The competitive growth of leaner dendrites has been frequently used to produce directionally solidified deposits in additive manufacturing or directionally solidified turbine blades [34,35]. These textured dendritic grains in the partially melted zone may then experience repetitive tempering by the subsequent thermal cycles for multipass welding. The Goss {110}<001> or rotated Goss {110}<110> texture components [36] along the fusion line region are very likely the results of formation and decomposition of austenite during the repeated multipass welding thermal cycles. Such an evolution of Goss {110} texture has been confirmed by others, such as Cerda et al. [37] and Bertolo et al. [38], for steels undergoing rapid heating at a higher rate than 150 °C/s. Welding heating is often faster than this.

Finally, the reason for the concentrated shear strain that has caused the crack initiation along the weld fusion boundary is most likely its being sandwiched between two stronger regions (the heat-affected zone and weld metal). Under a given load, the weld fusion line region will generate a greater displacement, as verified in Figure 8.

## 5. Conclusions

Flow-accelerated corrosion (FAC) preferentially attacks the downstream heat-affected zone of the root-pass weld in steam pipe systems. A detailed characterization identifies the fusion boundary as the initiation location for the attack. Experimental and computational methods confirm that the fusion boundary contains a greater fraction of grains with {110} slip planes aligned with the fusion boundary. The origin of the Goss texture is suggested as due to the directional epitaxial growth of dendrites during welding solidification. Due to the multiple welding thermal cycles, the fusion boundary undergoes repetitive austenite-to-ferrite phase transformations, leading to an increased proportion of grains with a Goss {110} texture. Chemical segregation across the weld fusion boundary explains the depletion of alloying elements. The synergistic effects of chemical segregation and easy slip systems may have contributed to the preferential initiation of FAC cracks along the root-pass weld fusion boundary, making it the weakest link for the attack in the steam pipe girth welds.

## Figures and Tables

**Table 1:** Chemical compositions of base metal and root-pass weld metal (wt.%).

	C	Mn	Ni	Mo	Si	Cr	Al	Cu	S	P	Fe
Base metal	0.09	1.41	0.038	0.097	0.27	0.02	0.02	0.01	0.003	0.01	Bal.
Weld metal	0.132	0.92	0.270	0.093	0.08	0.14	0.00	0.01	0.007	0.008	Bal.

**Table 2:** The grain sizes, grain boundary types (HAGB or LAGB) and their fractions, and precipitate volume fractions derived from the EBSD data shown in Figure 4.

Location	Grain Size	HAGB	LAGB	Cr_7_C_3_	Cr_23_C_6_	MoC	Mo_2_C	NbC
Fusion line	13.4 μm	78.70%	21.30%	0.30%	0.10%	0.70%	0.30%	0.10%
WM	13.0 μm	75.90%	24.10%	0.40%	0.10%	0.50%	0.40%	0.10%
HAZ	12.8 μm	76.10%	23.90%	0.20%	0.10%	0.50%	0.10%	0.10%

**Table 3:** Vicker’s hardness and Young’s modulus values measured from indentation tests and from EBSD data.

Location	Vicker’s Hardness (HV0.05)	Young’s Modulus from Indentation (GPa)	Young’s Modulus from EBSD (GPa)		
			X-Axis	Y-Axis	Z-Axis
Fusion Line	156.3 ± 7.59	183.97 ± 2.12	187.9	188.00	191.36
WM	164.6 ± 13.87	193.24 ± 7.43	193.89	202.13	196.47
HAZ	175.5 ± 16.05	198.42 ± 3.9	191.18	194.83	213.54

## Data Availability

The original contributions presented in this study are included in the article; further inquiries can be directed to the corresponding author.
